# Estimation of the Number of General Anesthesia Cases Based on a Series of Nationwide Surveys on Twitter during COVID-19 Pandemic in Japan: A Statistical Analysis

**DOI:** 10.3390/medicina57020153

**Published:** 2021-02-08

**Authors:** Yosuke Fujii, Hiroki Daijo, Kiichi Hirota

**Affiliations:** 1 Department of Human Stress Response Science, Institute of Biomedical Science, Kansai Medical University, Hirakata, Osaka 573-1010, Japan; yofujii-kyt@umin.ac.jp; 2 Department of Anesthesia, Takarazuka Daiichi Hospital, Takakazuka, Hyogo 665-0832, Japan; hirokidaijo412@gmail.com

**Keywords:** coronavirus disease 2019 (COVID-19), Twitter, general anesthesia, quadratic programming

## Abstract

*Background and objectives:* Coronavirus disease 2019 (COVID-19), caused by severe acute respiratory syndrome coronavirus 2 (SARS-CoV-2), has spread to more than 200 countries. In light of this situation, the Japanese Government declared a state of emergency in seven regions of Japan on 7 April 2020 under the provisions of the law. The medical care delivery system has been under pressure. Although various surgical societies have published guidelines on which to base their surgical decisions, it is not clear how general anesthesia has been performed and will be performed in Japan. *Materials and Methods:* One of the services provided by the social network service Twitter is a voting function—Twitter Polls—through which anonymous surveys were conducted. We analyzed the results of a series of surveys 17 times over 22 weeks on Twitter on the status of operating restrictions using quadratic programming to solve the mathematical optimizing problem, and public data provided by the Japanese Government were used to estimate the current changes in the number of general anesthesia performed in Japan. *Results:* The minimum number of general anesthesia cases per week was estimated at 67.1% compared to 2015 on 27 April 2020. The timeseries trend was compatible with the results reported by the Japanese Society of Anesthesiologists (correlation coefficient *r* = 0.69, *p <* 0.001*)*. *Conclusions:* The number of general anesthesia was reduced up to two-thirds during the pandemic of COVID-19 in Japan and was successfully quantitatively estimated using a quick questionnaire on Twitter.

## 1. Introduction

Coronavirus disease 2019 (COVID-19), caused by severe acute respiratory syndrome coronavirus 2 (SARS-CoV-2), was first reported in Wuhan, Hubei, China, and has since spread to more than 200 other countries around the world at the time of writing [1]. In light of this situation, the Japanese Government declared a state of emergency in seven regions of Japan on 7 April 2020 under the provisions of the law. This declaration was extended to the entire nation on 17 April 2020 and continued until 25 May 2020 [2]. The incidence of pneumonia from COVID-19 is considerably higher than from seasonal influenza, and the number of cases with no identifiable route of infection has increased rapidly [3,4]. The medical care delivery system has been under pressure [5]. Emphasis should be placed on maintaining medical care systems. Although various surgical societies have published guidelines on which to base their surgical decisions [6,7], it is not clear how general anesthesia has been performed [8,9].

Social networks such as Twitter are becoming a part of our society as various information is being accumulated on the web [10]. One of the services provided is a voting function called Twitter Polls. Using this function, anonymous surveys can be conducted on Twitter in Japan [11].

We analyzed the results of a series of surveys conducted on Twitter on the status of operating restrictions to estimate the number of general anesthesia performed during the COVID-19 pandemic in Japan. First, transition probability between the responses of the questionnaire, or the strength of restrictions, was estimated from the proportional data using quadratic programming to solve a mathematical optimizing problem. Second, public data provided by the Japanese Government and Markov Chain Monte Carlo simulation were used to estimate the current changes in the number of general anesthesia carried out in Japan. Finally, the time series trend of the estimation was compared with the results of the surveys sponsored by the Japanese Society of Anesthesiologists.

## 2. Materials and Methods

### 2.1. Twitter Surveys

The Twitter account @dajhiroki, which is run by a board-certified anesthesiologist and had approximately 1300 followers at the time of the survey, used Twitter Polls to conduct 24-hour surveys 17 times, which were held approximately 1 week apart from each other, from 13 March 2020 to 14 August 2020. During the period, the spread of COVID-19 had become a social problem in Japan.

The surveys used the same wording throughout the period in the form of a choice of 1 of the following 4 options in Japanese (Supplementary Information S1):

1. No surgical restrictions
2. Partial restrictions (more than half of the usual)
3. Extensive restrictions (less than half of the usual)
4. No scheduled surgery

### 2.2. Transition of Responses to the Survey

**Figure 1:**
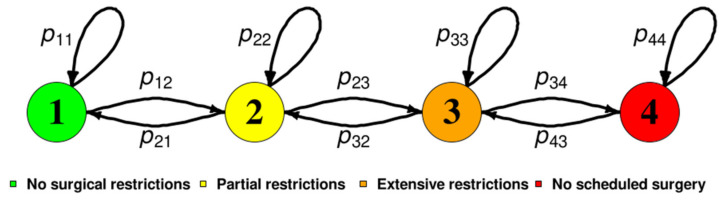
Transition scheme of the proportion of surveys. (1: Green) No surgical restrictions, (2: Yellow) partial restrictions, (3: Orange) extensive restrictions, and (4: Red) no scheduled surgery. $p_{ij}$ is the transition probability from response i to response j.

Suppose there were C=4 discrete categories of responses. The transition between the responses during the questionnaire period would be estimated (Figure 1). $P\left(t\right)$ was a 4×4 transition matrix whose elements $p_{ij}\left(t\right)$ showed the probability transition from the ith ($i=\left\{1,\;2,\;3,\;4\right\}$) category at time t ($t=\left\{1,\;2,\;\ldots ,\;16\right\}$) to the jth ($j=\left\{1,\;2,\;3,\;4\right\}$) category at time t+1. The transition matrix was

(1) $$P\left(t\right)=\left[\begin{array}{cccc} p_{11}\left(t\right) & p_{12}\left(t\right) & p_{13}\left(t\right) & p_{14}\left(t\right)\\ p_{21}\left(t\right) & p_{22}\left(t\right) & p_{23}\left(t\right) & p_{24}\left(t\right)\\ p_{31}\left(t\right) & p_{32}\left(t\right) & p_{33}\left(t\right) & p_{34}\left(t\right)\\ p_{41}\left(t\right) & p_{42}\left(t\right) & p_{43}\left(t\right) & p_{44}\left(t\right) \end{array}\right]$$

under the constraints of

(2) $$\left\{\begin{array}{c} \left\{p_{13}\left(t\right),\;p_{14}\left(t\right),\;p_{24}\left(t\right),p_{31}\left(t\right),p_{41}\left(t\right),p_{42}\left(t\right)\right\}=0\\ 0\leq \left\{p_{11}\left(t\right),p_{12}\left(t\right),\;p_{21}\left(t\right),\;p_{22}\left(t\right),p_{23}\left(t\right),\;p_{32}\left(t\right),\;p_{33}\left(t\right),p_{34}\left(t\right),\;p_{43}\left(t\right),\;p_{44}\left(t\right)\right\}\leq 1_{\;}\\ \;p_{11}\left(t\right)+p_{12}\left(t\right)=1_{\;}\\ p_{21}\left(t\right)+p_{22}\left(t\right)+p_{23}\left(t\right)=1_{\;}\\ p_{32}\left(t\right)+p_{33}\left(t\right)+p_{34}\left(t\right)=1_{\;}\\ p_{43}\left(t\right)+p_{44}\left(t\right)=1_{\;} \end{array}\right..$$

The number of the responses to the web questionnaire at time t, or *t*th Poll, was aggregated as the proportion $y_{i}\left(t\right)$, $i=\left\{1,\;2,\;3,\;4\right\}$. Here, $y_{i}\left(0\right)$ was assumed to be the data at t=0, or the status before the COVID-19 pandemic. All of the hospitals were not restricted as $y_{1}\left(0\right)=1$ and $y_{2}\left(0\right)=y_{3}\left(0\right)=y_{4}\left(0\right)=0$. When the individual transition was not available, it was not possible to estimate the transition matrix from individual transition data using the ordinary least square method. However, the quadratic programming method could estimate a transition matrix from proportional data [12]. A model of the relation between the actual response counts and estimated occurrence of $y_{i}\left(t\right)$ is described below with the error term $u_{j}\left(t\right)$:

(3) $$y_{j}\left(t+1\right)=\overset{4}{\underset{i}{\sum }}y_{i}\left(t\right)p_{ij}\left(t\right)+u_{j}\left(t\right).$$

This equation could be written in linear algebraic form as follows:

(4) $$y\left(t\right)=X\left(t\right)p\left(t\right)+u\left(t\right),$$

where

(5) $$y\left(t\right)={\left[y_{1}\left(t\right),\;y_{1}\left(t+1\right),\;y_{2}\left(t\right),y_{2}\left(t+1\right),y_{3}\left(t\right),\;y_{3}\left(t+1\right),\;y_{4}\left(t\right),y_{4}\left(t+1\right)\right]}^{\prime },$$

and

(6) $$X_{j}\left(t\right)=\left[\begin{array}{cccc} y_{1}\left(t-1\right) & y_{2}\left(t-1\right) & y_{3}\left(t-1\right) & y_{4}\left(t-1\right)\\ y_{1}\left(t\right) & y_{2}\left(t\right) & y_{3}\left(t\right) & y_{4}\left(t\right) \end{array}\right],$$

for $j=\left\{1,\;2,\;3,\;4\right\}$ so that

(7) $$X\left(t\right)=\left[\begin{array}{cccc} X_{1}\left(t\right) & 0 & 0 & 0\\ 0 & X_{2}\left(t\right) & 0 & 0\\ 0 & 0 & X_{3}\left(t\right) & 0\\ 0 & 0 & 0 & X_{4}\left(t\right) \end{array}\right],$$

(8) $$p\left(t\right)={\left[p_{1}\left(t\right),\;p_{1}\left(t+1\right),\;p_{2}\left(t\right),\;p_{2}\left(t+1\right),p_{3}\left(t\right),p_{3}\left(t+1\right),\;p_{4}\left(t\right),\;p_{4}\left(t+1\right)\right]}^{\prime },$$

and

(9) $$u\left(t\right)={\left[u_{1}\left(t\right),u_{1}\left(t+1\right),\;u_{2}\left(t\right),\;u_{2}\left(t+1\right),u_{3}\left(t\right),u_{3}\left(t+1\right),\;u_{4}\left(t\right),u_{4}\left(t+1\right)\right]}^{\prime }.$$

Estimation was performed minimizing the squared error term ${\left|u\left(t\right)\right|}^{2}$ as

(10) $${\left|u\left(t\right)\right|}^{2}={\left|y\left(t\right)-X\left(t\right)p\left(t\right)\right|}^{2}\;\;\;\;\;\;\;\;\;\;\;\;={\left|y\left(t\right)\right|}^{2}-2y{\left(t\right)}^{\prime }X\left(t\right)p\left(t\right)+p{\left(t\right)}^{\prime }X{\left(t\right)}^{\prime }X\left(t\right)p\left(t\right).$$

The objective of quadratic programming was to find a vector $p\left(t\right)$ that would minimize

(11) $$\frac{1}{2}p{\left(t\right)}^{\prime }X{\left(t\right)}^{\prime }X\left(t\right)p\left(t\right)-y{\left(t\right)}^{\prime }X\left(t\right)p\left(t\right),$$

subject to

(12) $$\mathrm{Ap}\left(t\right)\leq b,$$

where A is the coefficient matrix of $p\left(t\right)$ and b was a real vector corresponding to coefficients of equality or inequality for $p\left(t\right)$ denoted in Equation (2).

### 2.3. The Number of General Anesthesia under Restriction

**Figure 2:**
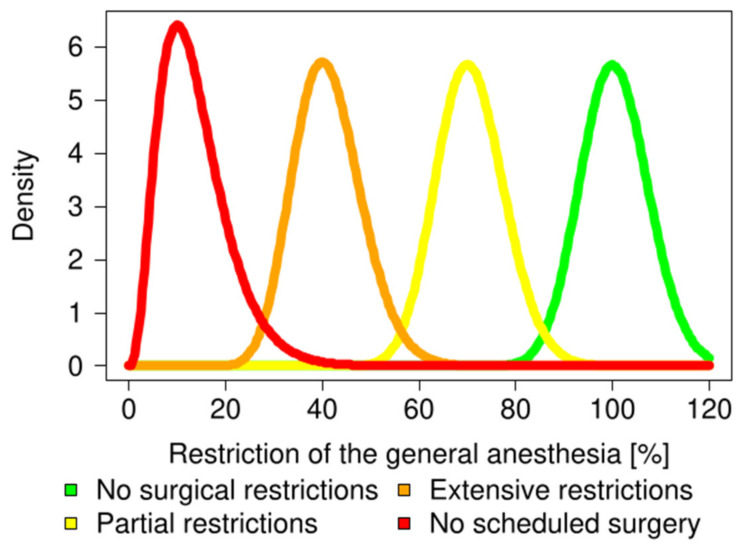
Distribution of the degree of restriction for each response group. The mean percentages of the restrictions were generated from the gamma distribution. No surgical restrictions (green), partial restrictions (yellow), extensive restrictions (orange), and no scheduled surgery (red) were generated from the gamma distribution ***Gamma***(*α*, *β*). The set of parameters for the gamma distribution, (*α*, *β*), were defined so that the respective median of the gamma distribution was 1.0 for “No surgical restrictions,” 0.7 for “Partial restrictions,” 0.4 for “Extensive restrictions,” and 0.1 for “No scheduled surgery.” The variance of the gamma distribution was 0.005.

The restriction rate of the number of general anesthesia was assumed for each response of the surveys: (1) “No surgical restrictions” was almost the same (100%) as the past statistics, (2) “Partial restrictions” was about 70% restriction rate compared to the past statistics, (3) “Extensive restrictions” was about 40% restriction rate compared to the past statistics, and (4) “No scheduled surgery” was about 10% restriction rate compared to the past statistics (Figure 2). Under such assumption, the restriction rate for each hospital h of the restriction status i at week t, $x_{t,\;i,h}$ was generated from the gamma distribution

(13) $$\mathrm{Gamma}\left(x;\alpha ,\;\beta \right)=\frac{\beta^{\alpha }x^{\alpha -1}e^{-\beta x}}{\int_{0}^{\infty }t^{\alpha -1}e^{-t}dt},$$

where α>0 and β>0. Their parameters $\left(\alpha_{i},\;\beta_{i}\right)$,$\;i=\left\{1,\;2,\;3,\;4\right\}$ were defined so that the median of the gamma distribution corresponding to the restriction status was 1.0 for “No surgical restrictions,” 0.7 for “Partial restrictions,” 0.4 for “Extensive restrictions,” and 0.1 for “No scheduled surgery,” and the variance of the gamma distribution was 0.005, respectively (Figure 2). The number of general anesthesia performed at hospital h in the tth week, $R_{h,\;t}$, was generated by Markov Chain Monte Carlo simulation as follows:

(14) $$x_{t,i,h}∼\mathrm{Gamma}\left(\alpha_{i},\beta_{i}\right),$$

(15) $$R_{h,t}=N_{h}x_{t,i,h},$$

where $N_{h}$ is the reference number of the general anesthesia performed at hospital h (data available from e-Stat).

The response to the survey for each hospital at t=1 was randomly allocated to the ith response according to $y_{i}\left(1\right)$ and its status transitioned to the jth response by $p_{ij}\left(t\right)$.

For sensitivity analysis, two other scenarios were considered. An optimistic scenario assumed relatively high performance in operating rooms, with an average of 80% for “2. Partial restrictions,” 50% for “3. Extensive restrictions,” and 25% for “4. No scheduled surgery.” A pessimistic scenario assumed extremely low performance, with an average of 50% for “2. Partial restrictions,” 30% for “3. Extensive restrictions,” and 3% for “4. No scheduled surgery.”

Analysis and estimation were performed using the quadprog package (version 1.5.8) within the R programming language (version 3.4.4). Point estimates and their statistical uncertainty were presented as 95% confidential intervals (CI) with 1000 iterations. The data were obtained from the Japan official statistics portal site (e-Stat), a public database in Japan (https://www.e-stat.go.jp/stat-search/file-download?statInfId=000031682822&fileKind=0 accessed on 8 February 2021), and were preprocessed for analysis. This dataset contained the labels of “row id,” “hospital id,” “hospital name,” “total number,” “with surgery,” “with chemotherapy,” “with radiation therapy,” “with ambulance transfer,” “either,” “general anesthesia,” and their percentages in Japanese. The number of general anesthesia was defined as general anesthesia performed with an open drop system or closed circulation system in the Japanese healthcare system. The columns “hospital name” and “general anesthesia” were extracted from the XLS file for preprocessing. The code and preprocessed dataset are available online (https://github.com/yfujii08/covid19generalanesthesia accessed on 8 February 2021) and available upon request from the corresponding author.

### 2.4. Nationwide Report of the Estimation of the Number of General Anesthesia from the Japanese Society of Anesthesiologists (JSA)

The JSA searched the extent of the restriction of general anesthesia by questionnaires. They sent questionnaires to all the authorized institutes (*n* = 1415) from 23 April 2020 approximately every week. The proportion of the number of general anesthesia compared to the previous year was estimated and disclosed to only the members of JSA (https://anesth.or.jp/img/upload/ckeditor/files/2004_07_08.pdf accessed on 8 February 2021). The JSA asked its authorized members in a questionnaire whether they had prepared a special operating room for the patients with COVID-19, how many surgeries were performed in the week when they responded to the questionnaire, and how many surgeries were performed in the previous year, and they reported the regional difference. The results of the percentage of surgeries compared to the previous year were used. The similarity between our estimation and the JSA results was measured with a correlation coefficient. The estimated values for the Twitter Polls and JSA report could not be directly compared because the timepoints, or dates, of Twitter Polls and the JSA report were different. The unpaired values were linearly interpolated and the correlation coefficient, r, was defined as

(16) $$r\left(f_{Polls},\;f_{JSA}\right)=\frac{\sum \left(f_{Polls}-{\bar{f}}_{Polls}\right)\left(f_{JSA}-{\bar{f}}_{JSA}\right)}{\sqrt{\sum {\left(f_{Polls}-{\bar{f}}_{Polls}\right)}^{2}}\sqrt{\sum {\left(f_{JSA}-{\bar{f}}_{JSA}\right)}^{2}}},$$

where $f_{Polls}$ is the estimation from Twitter Polls with the interpolated values corresponding to the date of the JSA report. ${\bar{f}}_{Polls}$ is the mean of $f_{Polls}$, and analogously for $f_{JSA}$.

### 2.5. Ethics

This study is exempted from institutional review board approval because this study does not contain human participants or research material derived from human participants.

## 3. Results

From 13 March 2020 to 14 August 2020, 17 survey responses were solicited approximately weekly via a Twitter account (@dajhiroki) in the Japanese language, with the number of responses received ranging from 47 to 288 (Supplementary Information S1). 

In the public database (e-Stat), 3501 hospitals were registered as facilities that provide general anesthesia. Of the 3311 hospitals that reported the number of general anesthesia cases, 1989 performed more than 100 cases of general anesthesia.

**Figure 3:**
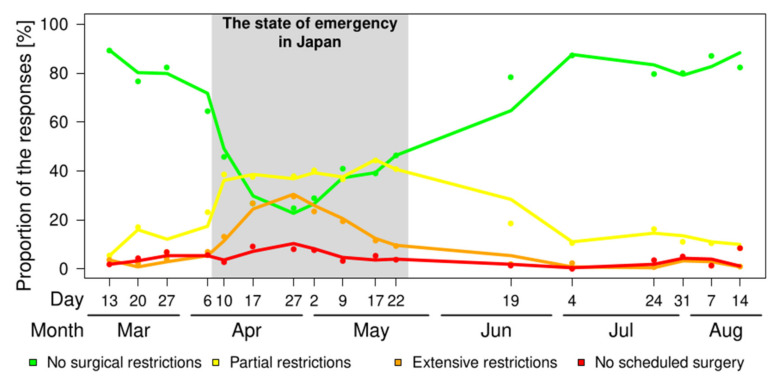
The transition of the proportion of responses to the survey. A solid line was an estimated transition and dots were actual data.

From the survey conducted in the first week (13 March 2020), 89.2% had no surgical restrictions, but this proportion decreased to 24.7% in the survey conducted in the seventh week (27 April 2020) and slightly recovered to 38.9% (17 May 2020). The proportion recovered to 87.2% (4 July 2020), and finally recovered to 82.3% at the end of the questionnaire (14 August 2020). The proportions of partial restrictions, extensive restrictions, and no scheduled surgeries were 5.4%, 3.6%, and 1.8% (13 March 2020); 37.8%, 29.5%, and 8.0% (27 April 2020); 44.2%, 11.5%, and 5.3% (17 May 2020); and 8.4%, 0.9%, and 8.4% (14 August 2020), respectively (Figure 3). The estimated proportion of each response and its transition are shown in Figure 3.

**Figure 4:**
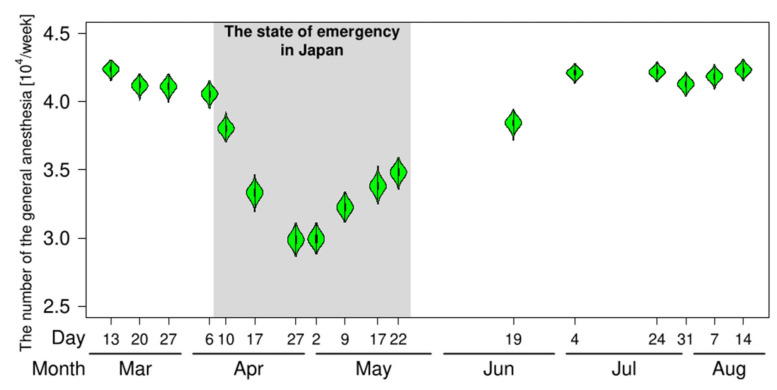
Estimation of the number of general anesthesia in Japan from the survey results. The violin plots show the distribution of the estimated number of general anesthesia performed at 1989 hospitals in Japan.

The number of general anesthesia performed decreased as the restrictions increased (Figure 4). Before COVID-19, 4.45 × 10^4^ cases of general anesthesia were performed per week in Japan. The number of general anesthesia (10^4^ cases per week unit) was estimated to be 4.24 [95% CI: 4.18, 4.29] in the first-week survey (13 March 2020), 2.99 [2.91, 3.07] in the seventh-week survey (27 April 2020) and 4.23 [4.18, 4.28] in the last week survey (14 August 2020).

**Figure 5:**
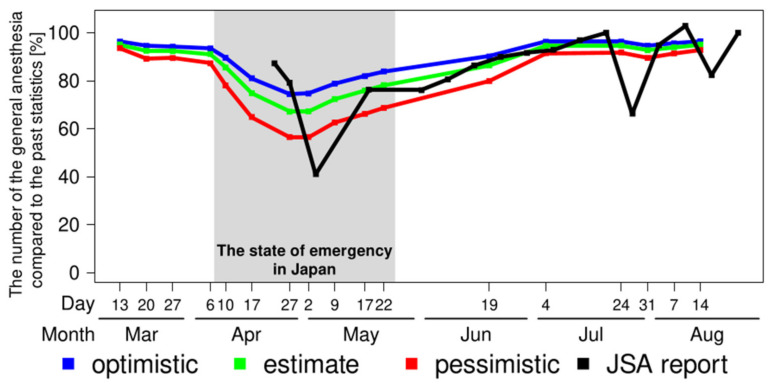
Estimation of the number of general anesthesia in Japan from the results of the surveys for the three scenarios as a sensitivity analysis and comparison between the estimation of the number of the general anesthesia and the report from the Japanese Society Anesthesiologists (JSA). The JSA reported the results of the estimated number of general anesthesia from 1415 authorized institutes from 23 April 2020.

Sensitivity analysis showed that the number of general anesthesia performed decreased according to the intensity of the restrictions (Figure 5). From the first week of the survey (13 March 2020), the optimistic scenario had a median of 4.29 × 10^4^ cases/week (96.3% compared to 2015), while the pessimistic scenario had a median of 4.17 × 10^4^ cases/week (93.7%). From the seventh week of the survey (27 April 2020), the optimistic scenario median was 3.32 × 10^4^ cases/week (74.5%), while that of the pessimistic scenario was 2.52 × 10^4^ cases/week (56.5%), respectively. From the last week of the survey (14 August 2020), the optimistic scenario median was 4.30 × 10^4^ cases/week (96.5%), while that of the pessimistic scenario was 4.13 × 10^4^ cases/week (92.9%). The maximum difference between optimistic and pessimistic scenarios was 1.32-fold on 2 May 2020.

The comparison between the estimation of this survey and the nationwide reports from the surveys of the JSA is shown in Figure 5. The data before the end of April are missing because the questionnaire started on 23 April 2020. The JSA data show a gradual decrease in the number of general anesthesia compared to the previous year, with 87.3% on 23 April, 79.2% on 27 April, and 41.1% on 4 May, respectively. As JSA pointed out, there was a drastic decrease on 3 May and 25 July. These declines came from sequential national holidays. The former decline resulted from “Golden Week” in Japan, with an extra two holidays compared to the same week of the previous year, while the latter resulted from sequential holidays designated for the Olympic year, adding two holidays in the week. After that, the number of general anesthesia cases, as well as the estimation based on this survey, gradually recovered. There was significant correlation between estimates and JSA report (correlation coefficient *r* = 0.69, *p* < 0.001).

## 4. Discussion

It is difficult to adequately control for the level of limitation of usual surgical care during the expansion of COVID-19. It is determined by adherence to official guidelines, restrictions on medical supplies and medical staff, and the level of need for surgery. Still, because circumstances can change quickly, it is often determined by reference to the extent of restrictions at a medical facility other than the home facility.

The staged approach—recommended by the Centers for Medicare and Medicaid Services (CMS) and the American College of Surgeons (ACS)—was used as a guide to determine how to perform surgery in situations where the preservation of ventilators and personal protective equipment is necessary and where the ICU has been compromised or is expected to be compromised soon.

The impact of COVID-19 on general anesthesia has not been quantifiably evaluated [8,9] but new evidence is emerging. A nationwide questionnaire survey in Turkey demonstrated that 62.1% of the responders stopped elective surgeries after the first case was reported in Turkey (11 March 2020) [13]. A cross-sectional analysis in northwest England reported that the general anesthesia rates for cesarean section were significantly reduced from 7.7% to 3.7% due to the COVID-19 pandemic [14]. The largest, latest nationwide survey from the COVIDSurg group showed that 72.3% of surgeries were canceled or postponed during the 12-week peak of disruption caused by the COVID-19 pandemic [15]. They reported a 73.2% cancellation rate for Japan [15], which was much larger than our estimates. However, their estimates were based on another survey which reported that the Japanese surgical volume ranged from 7.7 to 16.6 million [16], which would include regional anesthesia. Regarding cancer surgery, which should be assumed to be performed under general anesthesia, Japanese cancellation rates were estimated as 30.1 ranging from 17.1 to 44.9. This quantification is compatible with our results.

Using a series of surveys conducted through Twitter Polls, we were able to quickly determine the extent of the restrictions on surgery at medical facilities across the country about one month ahead of the survey by the JSA. By periodically soliciting responses to the same survey on Twitter, we were able to estimate the extent to which operations would be restricted nationally over time.

Our methodology in this study is an estimation method that uses mathematical analysis. In this study, we used public data from a database operated by the Japanese Government. The number of hospitals accredited by the JSA is 1415. However, in this study, 1989 hospitals that perform at least 100 surgeries per year under general anesthesia were used. The number of operations performed at each hospital varies mainly according to the size of the hospital. On the other hand, since neither the size of the hospitals to which the survey respondents belonged nor the changes in the number of operations were known, we used mathematical analysis to calculate the number of operations.

There are several limitations to this study. First, the number and type of survey responses is a potential limitation. The number of anesthesiologists using Twitter is not sufficient to obtain the definitive estimation, and there may be some bias in terms of the responses from those that do use it. Responses were low initially, with 47 in the early stages of the survey period. In the latter half of the survey period, responses increased to 288. The growing penetration of the surveys and increasing familiarity with the issue of operating room restrictions may have contributed to an increase in participation, but this may have been biased. Second, we calculated the number of surgeries as 100% for the criteria “No surgical restrictions,” 70% for “Partial restrictions,” 40% for “Extensive restrictions,” and 10% for “No scheduled surgery.” In the sensitivity analysis, two scenarios were assumed, but the number of general anesthesia cases performed could have led to a difference of approximately 1.32-fold following the survey period. Because it is difficult to estimate the accurate extent of restrictions, intuitive values were adopted. The results need to be compared with the descriptive statistics after the era of COVID-19.

## 5. Conclusions

In conclusion, we analyzed the results of a series of surveys on Twitter on the status of operating restrictions during the COVID-19 pandemic era using quadratic programming to solve a mathematical optimizing problem, and public data provided by the Japanese Government were used to estimate and compare the current changes in the number of general anesthesia carried out in Japan. The number of general anesthesia was reduced up to two-thirds during the COVID-19 pandemic in Japan and was successfully quantitatively estimated using a quick questionnaire on Twitter. The timeseries trend of the estimation was compared with the results of the surveys sponsored by the JSA. The COVID-19 pandemic is ongoing worldwide. In this study, we applied this methodology to estimate the number of surgeries with anesthesia during the so-called first wave in one region, Japan. However, this methodology can be applied to the analysis of several previous and current epidemics and can be used in fields other than the estimation of the number of surgeries. Thus, it is considered to be a technique with rich applicability.

## Data Availability

Publicly available datasets were analyzed in this study. This data can be found here: [https://github.com/yfujii08/covid19generalanesthesia]. The data and code presented in this study are available on request from the corresponding author.

## Supplementary Materials

The following are available online at https://www.mdpi.com/1010-660X/57/2/153/s1, Supplementary Information S1. Twitter Polls conducted by @ dajhiroki.
