# Supplementary material for: Estimation of the Number of General Anesthesia Cases Based on a Series of Nationwide Surveys on Twitter during COVID-19 Pandemic in Japan: A Statistical Analysis
Source: Medicina (Kaunas). 2021 Feb 8;57(2):153. doi: 10.3390/medicina57020153 (PMC7915187; doi:10.3390/medicina57020153)
Supplement: Supplementary file 1 [file medicina-57-00153-s001.pdf]

Supplementary Information S1

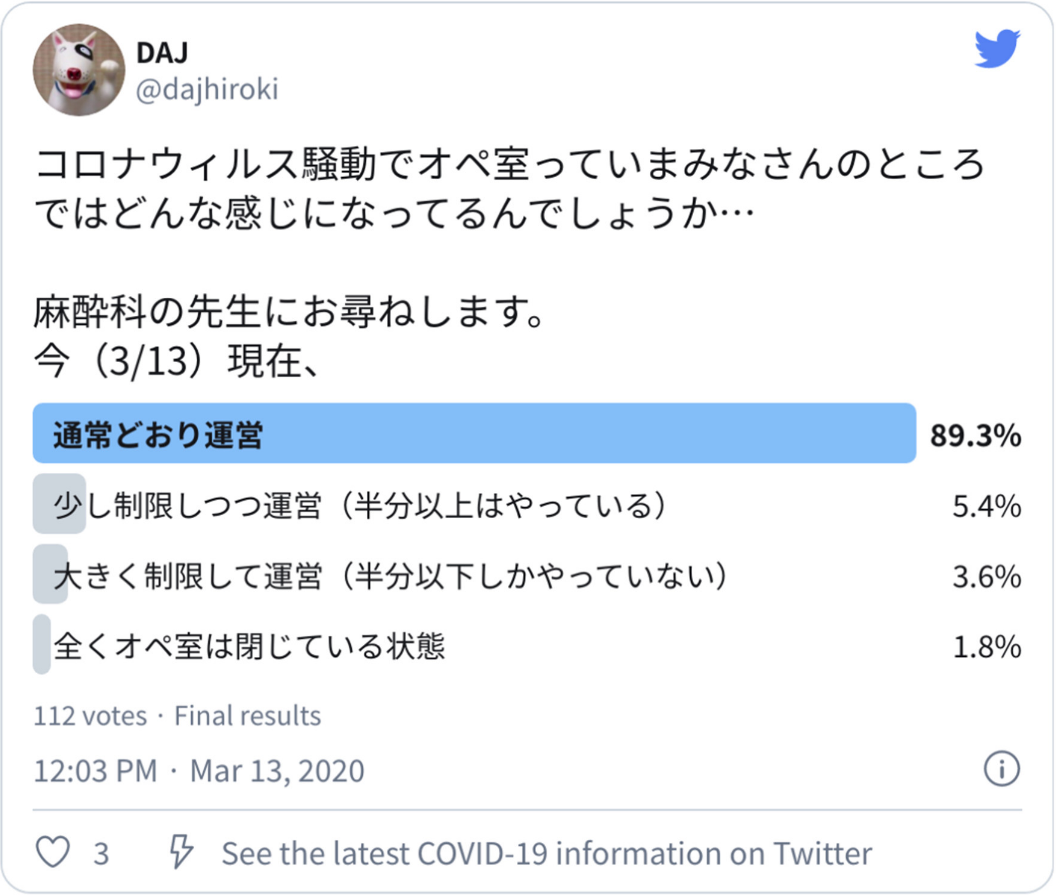

DAJ @dajhiroki

How is your operation room in coronavirus pandemic?

I would ask anesthesiologists the question. As of 13 March (3/13),

Operating as usual	89.3%
Operating with partial restrictions (more than half of the usual)	5.4%
Operating with extensive restrictions (less than half of the usual)	3.6%
Operation room is completely closed.	1.8%

https://twitter.com/dajhiroki/status/1238299708059074562

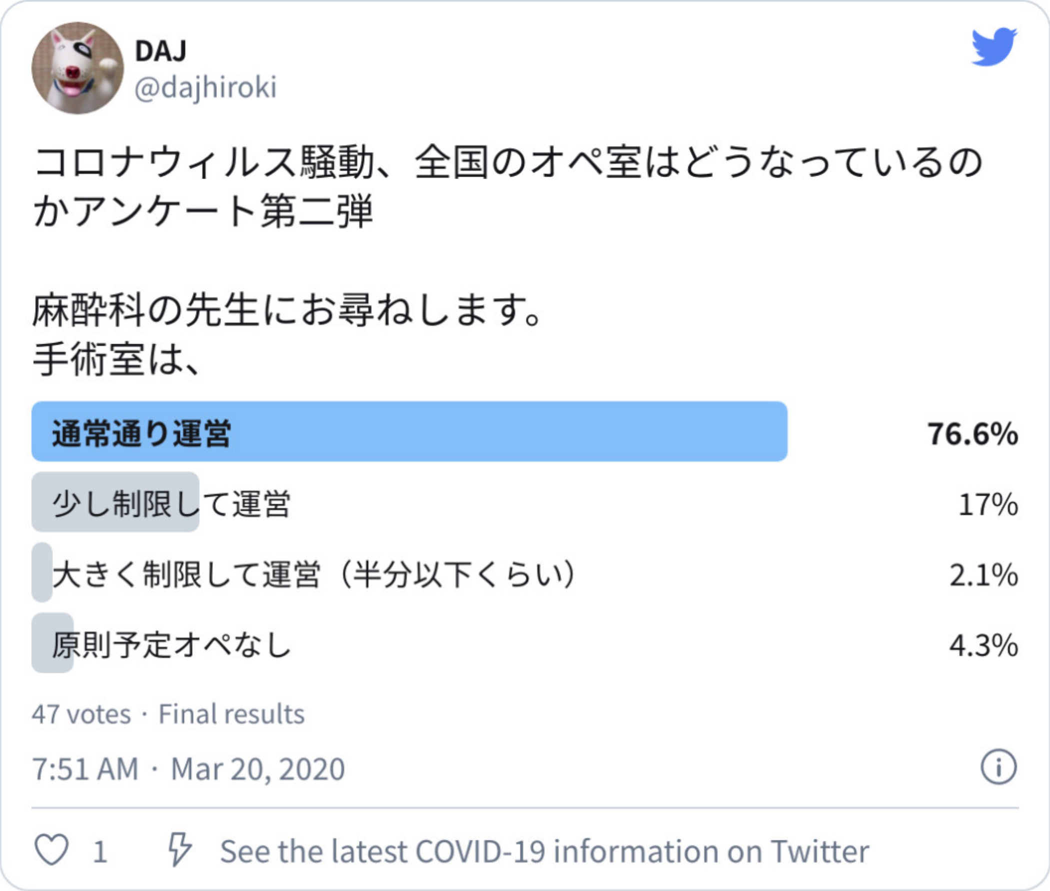

DAJ @dajhiroki

How is your operation room in coronavirus pandemic? The second trial.

I would ask anesthesiologists the question. Operation room performs…

Operating as usual	76.6%
Operating with partial restrictions	17%
Operating with extensive restrictions (approximately less than half)	2.1%
As a general rule, no surgery	4.3%

https://twitter.com/dajhiroki/status/1240772960924794880

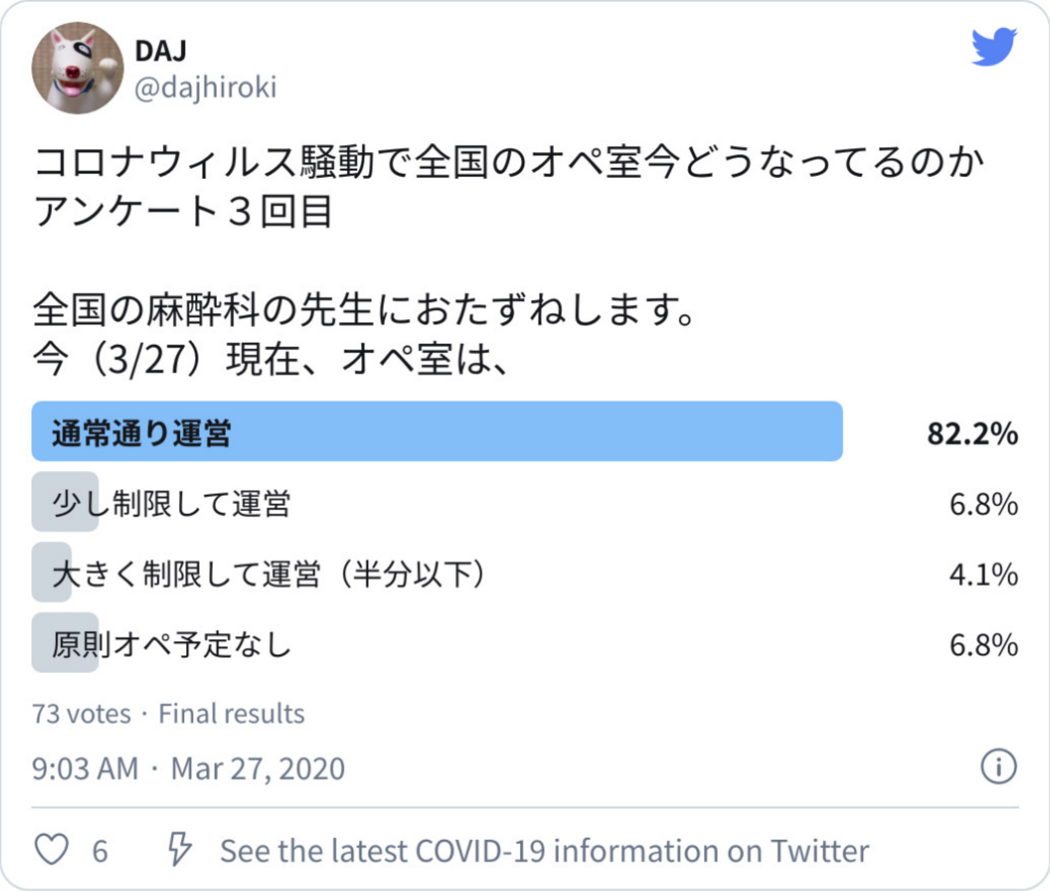
DAJ
@dajhiroki

コロナウィルス騒動で全国のオペ室今どうなってるのかアンケート３回目

全国の麻酔科の先生におたずねします。
今（3/27）現在、オペ室は、

通常通り運営	82.2%
少し制限して運営	6.8%
大きく制限して運営（半分以下）	4.1%
原則オペ予定なし	6.8%

73 votes · Final results

9:03 AM · Mar 27, 2020

6 See the latest COVID-19 information on Twitter

DAJ @dajhiroki

How is your operation room in coronavirus pandemic? The third trial.

I would ask anesthesiologists the question. As of 27 March (3/27), operation room performs…

Operating as usual	82.2%
Operating with partial restrictions	6.8%
Operating with extensive restrictions (less than half)	4.1%
As a general rule, no surgery	6.8%

https://twitter.com/dajhiroki/status/1243327743040745472

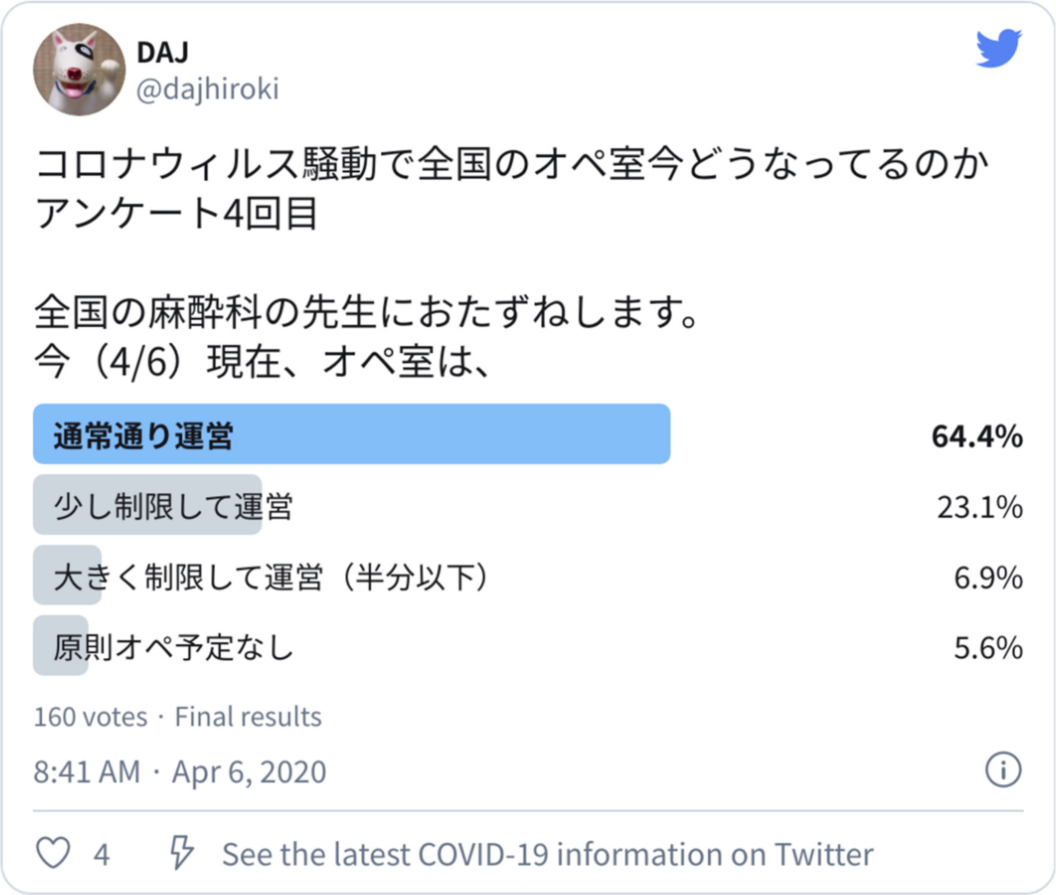

DAJ @dajhiroki

How is your operation room in coronavirus pandemic? The fourth trial.

I would ask anesthesiologists the question. As of 6 April (4/6), operation room performs…

Operating as usual	64.4%
Operating with partial restrictions	23.1%
Operating with extensive restrictions (less than half)	6.9%
As a general rule, no surgery	5.6%

https://twitter.com/dajhiroki/status/1246946140630552576

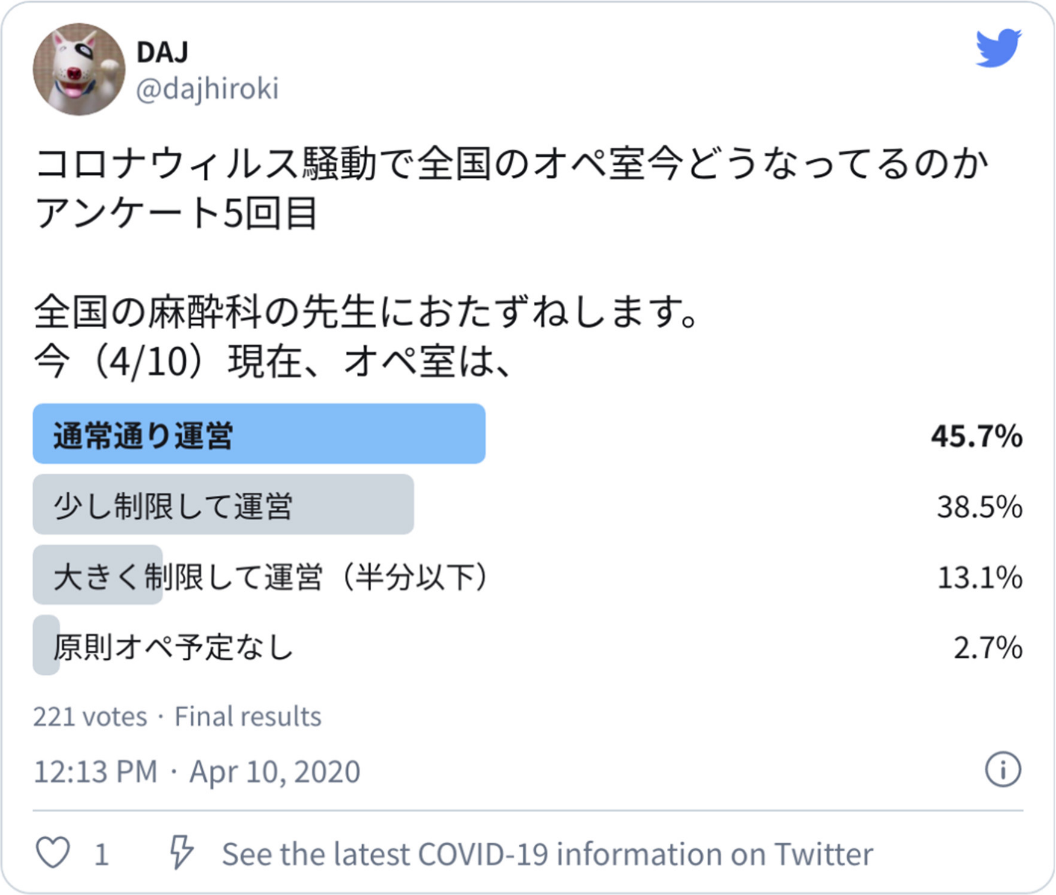

DAJ @dajhiroki

How is your operation room in coronavirus pandemic? The fifth trial.

I would ask anesthesiologists the question. As of 10 April (4/10), operation room performs…

Operating as usual	45.7%
Operating with partial restrictions	38.5%
Operating with extensive restrictions (less than half)	13.1%
As a general rule, no surgery	2.7%

https://twitter.com/dajhiroki/status/1248448870377373697

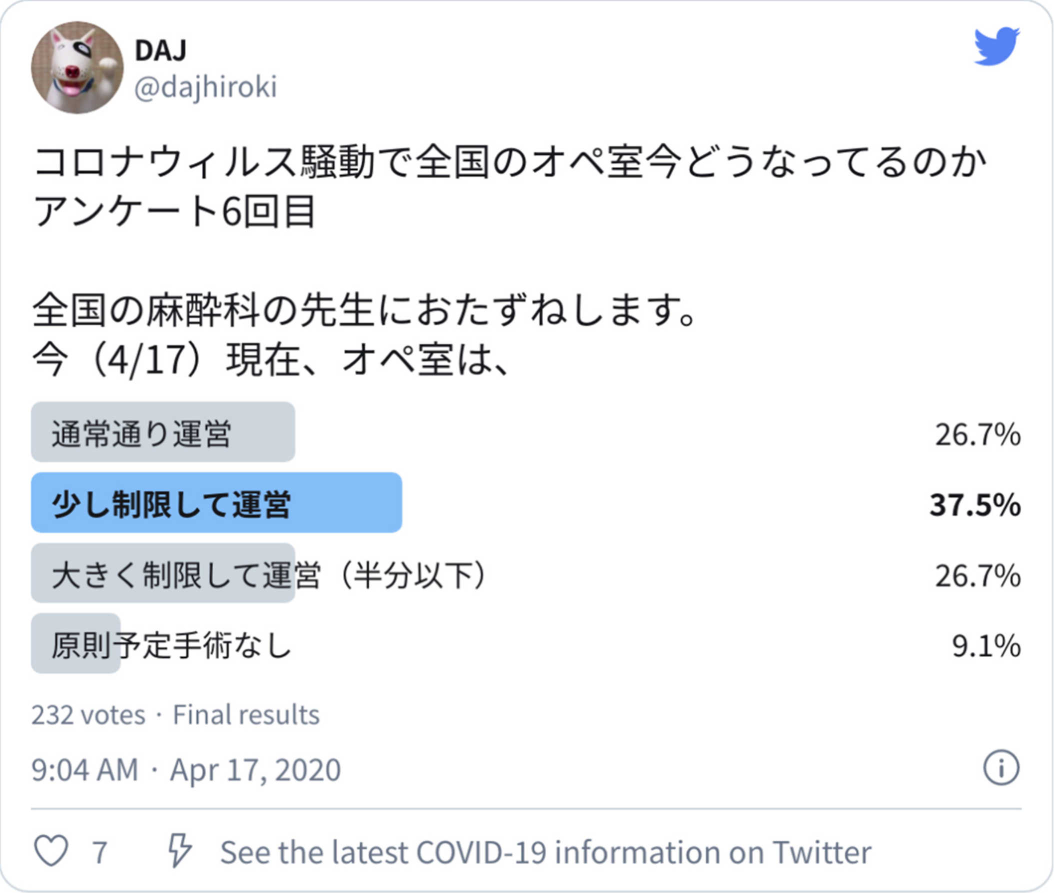
DAJ
@dajhiroki

コロナウィルス騒動で全国のオペ室今どうなってるのか
アンケート6回目

全国の麻酔科の先生におたずねします。
今（4/17）現在、オペ室は、

通常通り運営	26.7%
**少し制限して運営**	**37.5%**
大きく制限して運営（半分以下）	26.7%
原則予定手術なし	9.1%

232 votes · Final results

9:04 AM · Apr 17, 2020

7 See the latest COVID-19 information on Twitter

DAJ @dajhiroki

How is your operation room in coronavirus pandemic? The sixth trial.

I would ask anesthesiologists the question. As of 17 April (4/17), operation room performs…

Operating as usual	26.7%
Operating with partial restrictions	37.5%
Operating with extensive restrictions (less than half)	26.7%
As a general rule, no surgery	9.1%

https://twitter.com/dajhiroki/status/1250938192334815234

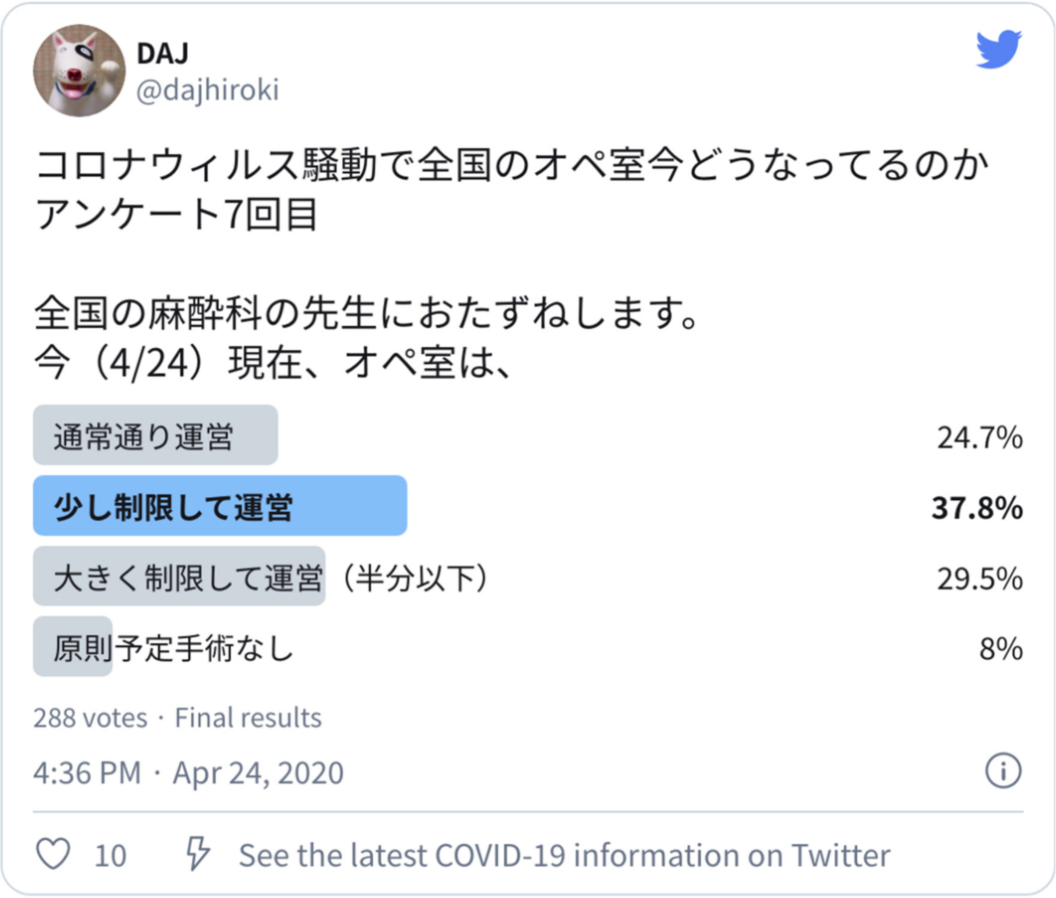

DAJ @dajhiroki

How is your operation room in coronavirus pandemic? The seventh trial.

I would ask anesthesiologists the question. As of 24 April (4/24), operation room performs…

Operating as usual	24.7%
Operating with partial restrictions	37.8%
Operating with extensive restrictions (less than half)	29.5%
As a general rule, no surgery	8%

https://twitter.com/dajhiroki/status/1253588554103402498

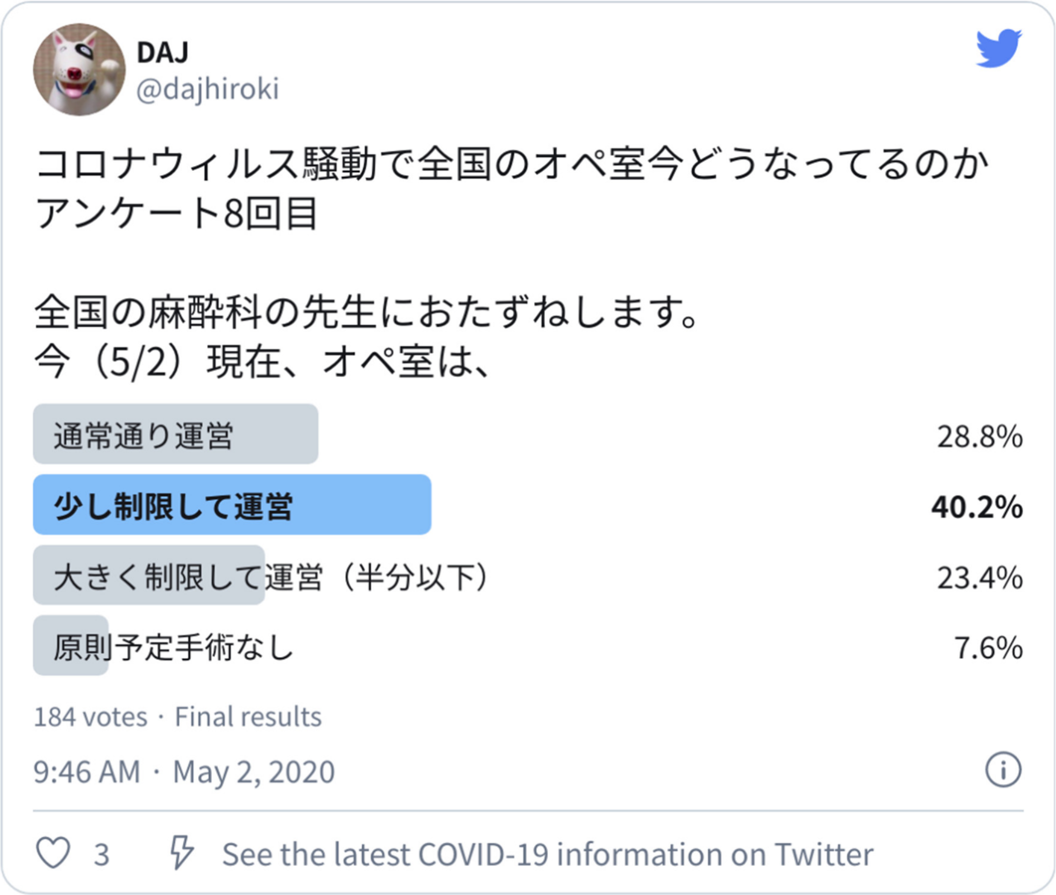

DAJ @dajhiroki

How is your operation room in coronavirus pandemic? The eighth trial.

I would ask anesthesiologists the question. As of 2 May (5/2), operation room performs…

Operating as usual	28.8%
Operating with partial restrictions	40.2%
Operating with extensive restrictions (less than half)	23.4%
As a general rule, no surgery	7.6%

https://twitter.com/dajhiroki/status/1256384655458107394

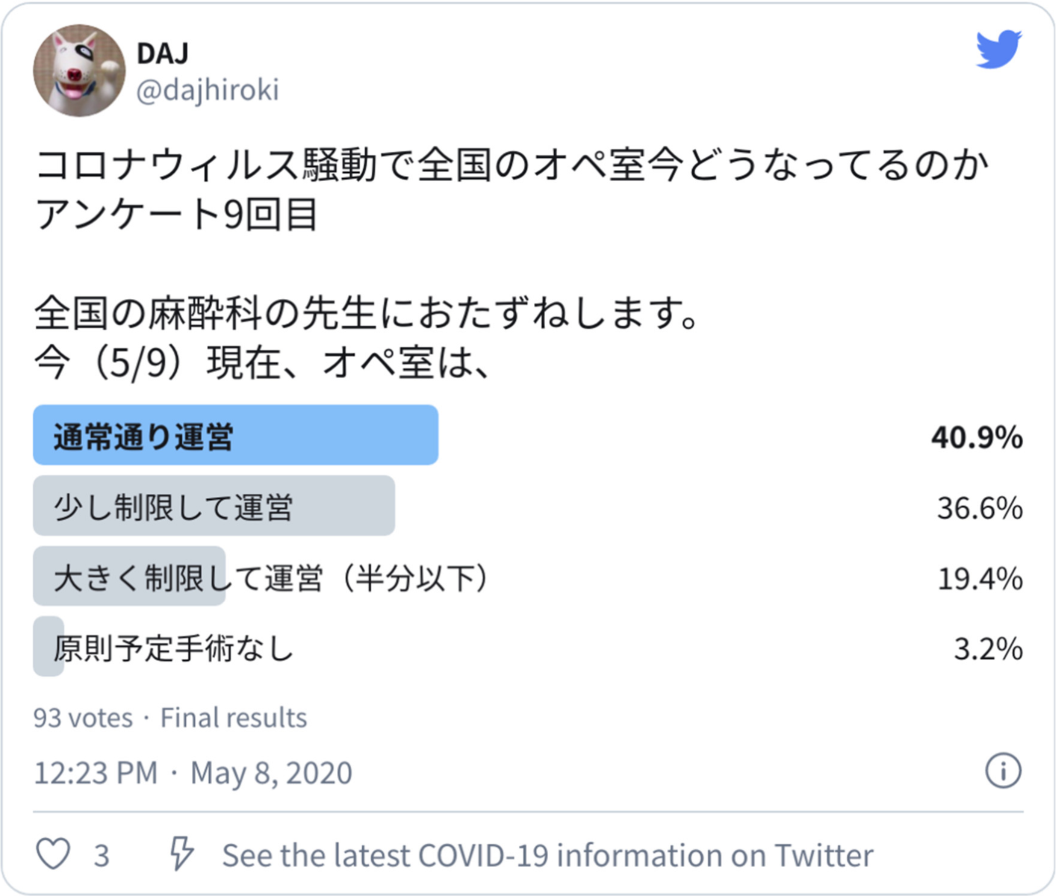

DAJ
@dajhiroki

コロナウィルス騒動で全国のオペ室今どうなってるのか
アンケート9回目

全国の麻酔科の先生におたずねします。
今（5/9）現在、オペ室は、

**通常通り運営**	**40.9%**
少し制限して運営	36.6%
大きく制限して運営（半分以下）	19.4%
原則予定手術なし	3.2%

93 votes · Final results

12:23 PM · May 8, 2020

3 See the latest COVID-19 information on Twitter

DAJ @dajhiroki

How is your operation room in coronavirus pandemic? The ninth trial.

I would ask anesthesiologists the question. As of 9 May (5/9), operation room performs…

Operating as usual	40.9%
Operating with partial restrictions	36.6%
Operating with extensive restrictions (less than half)	19.4%
As a general rule, no surgery	3.2%

https://twitter.com/dajhiroki/status/1258598314494136320

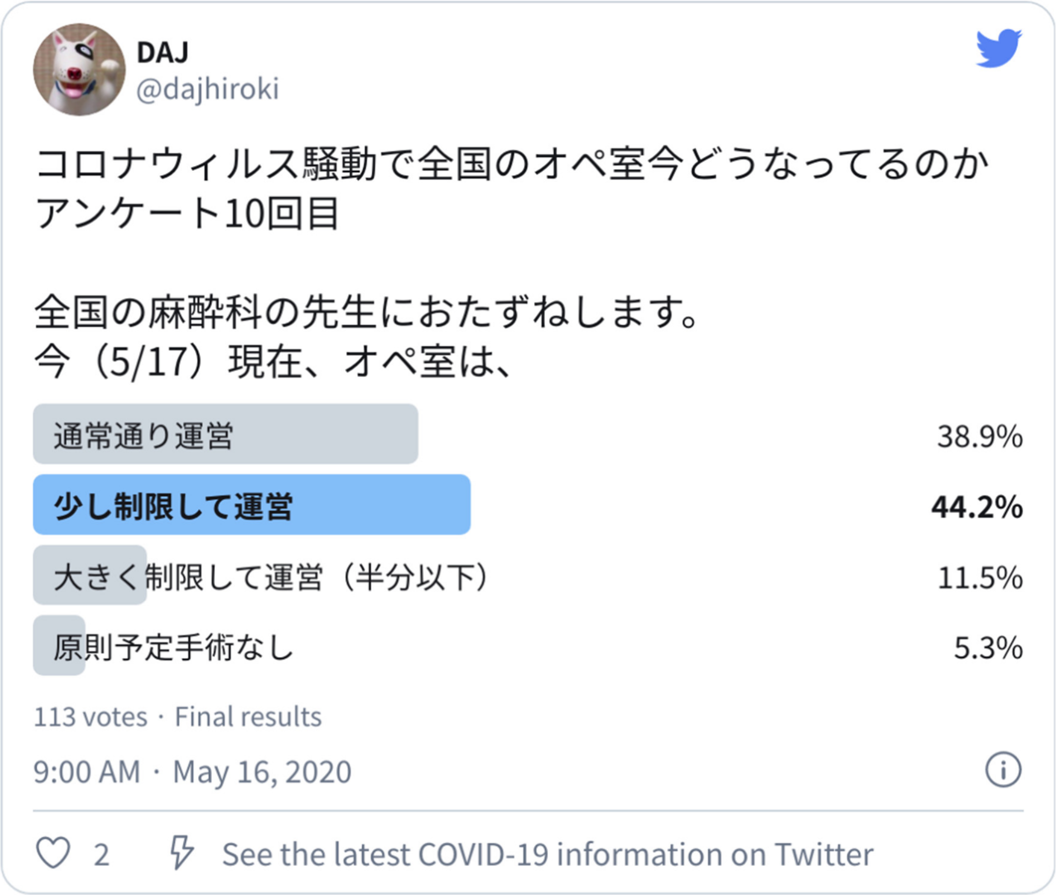

DAJ @dajhiroki

How is your operation room in coronavirus pandemic? The tenth trial.

I would ask anesthesiologists the question. As of 17 May (5/17), operation room performs…

Operating as usual	38.9%
Operating with partial restrictions	44.2%
Operating with extensive restrictions (less than half)	11.5%
As a general rule, no surgery	5.3%

https://twitter.com/dajhiroki/status/1261446399377301504

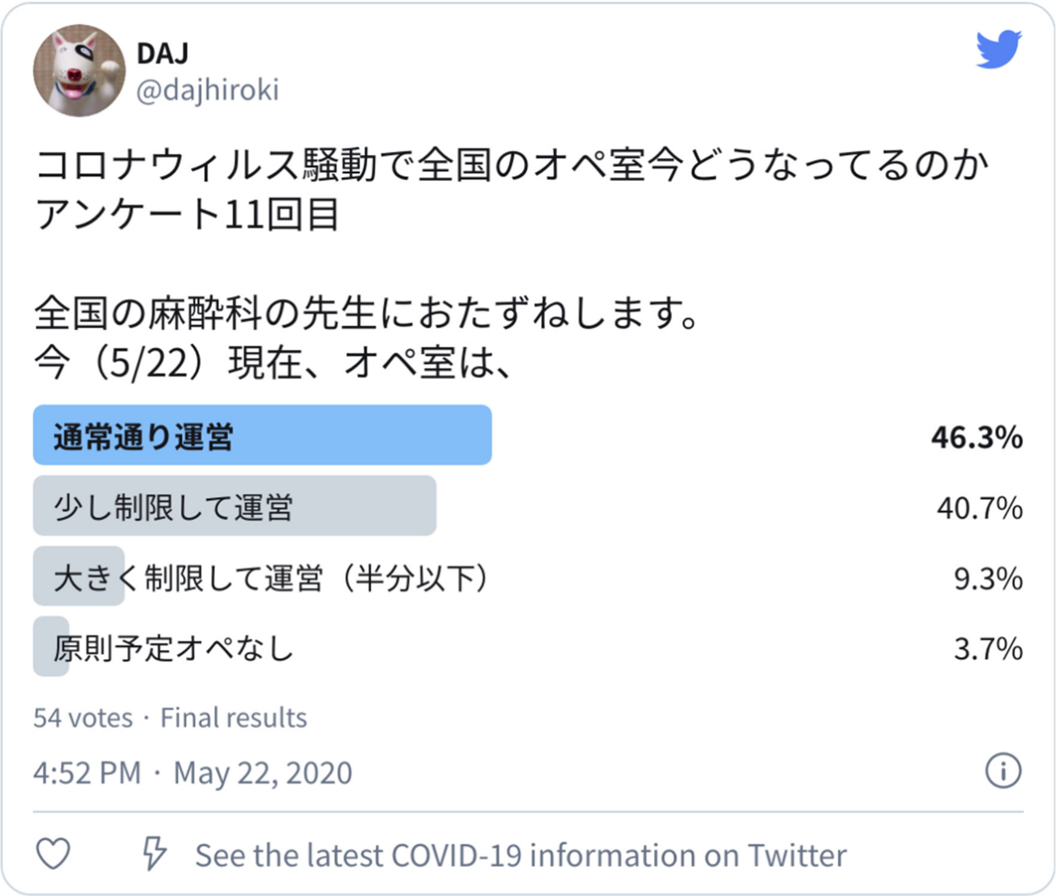
DAJ
@dajhiroki

コロナウィルス騒動で全国のオペ室今どうなってるのかアンケート11回目

全国の麻酔科の先生におたずねします。
今（5/22）現在、オペ室は、

**通常通り運営**	**46.3%**
少し制限して運営	40.7%
大きく制限して運営（半分以下）	9.3%
原則予定オペなし	3.7%

54 votes · Final results

4:52 PM · May 22, 2020

See the latest COVID-19 information on Twitter

DAJ @dajhiroki

How is your operation room in coronavirus pandemic? The eleventh trial.

I would ask anesthesiologists the question. As of 22 May (5/22), operation room performs…

Operating as usual	46.3%
Operating with partial restrictions	40.7%
Operating with extensive restrictions (less than half)	9.3%
As a general rule, no surgery	3.7%

https://twitter.com/dajhiroki/status/1263739620321157120

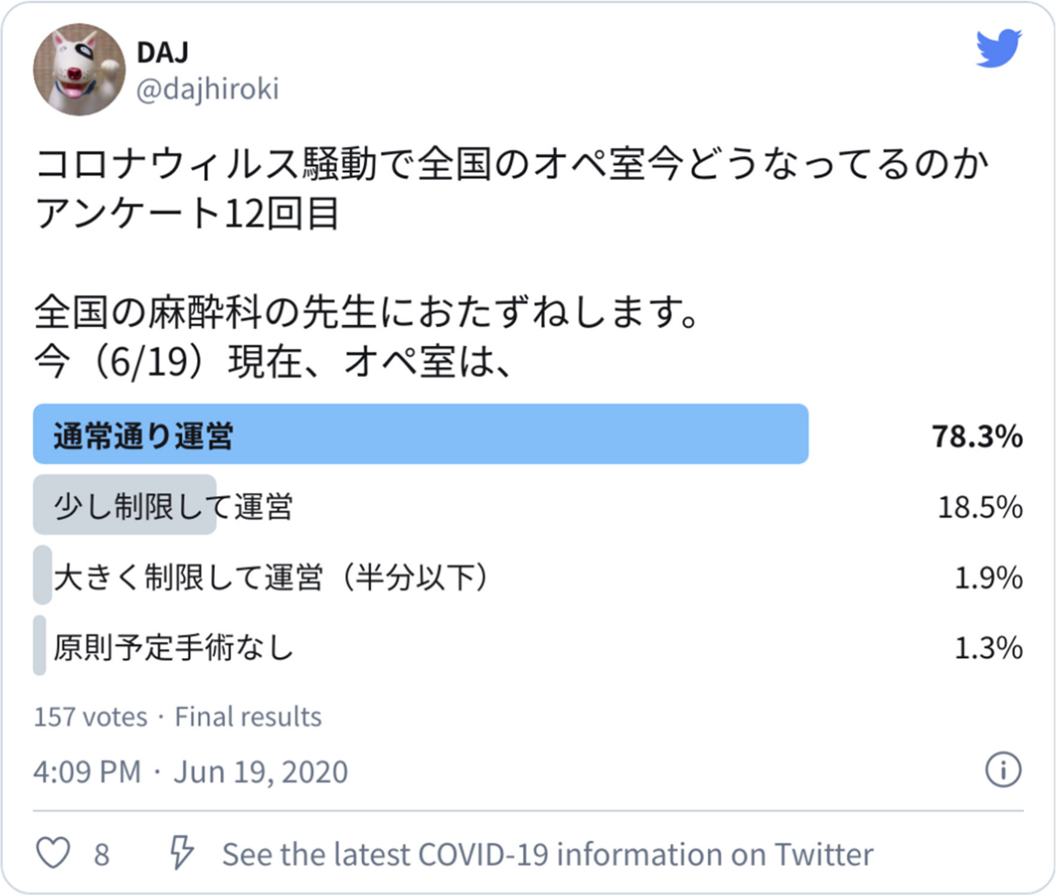

DAJ @dajhiroki

How is your operation room in coronavirus pandemic? The twelfth trial.

I would ask anesthesiologists the question. As of 19 June (6/19), operation room performs…

Operating as usual	78.3%
Operating with partial restrictions	18.5%
Operating with extensive restrictions (less than half)	1.9%
As a general rule, no surgery	1.3%

https://twitter.com/dajhiroki/status/1273875534611574785

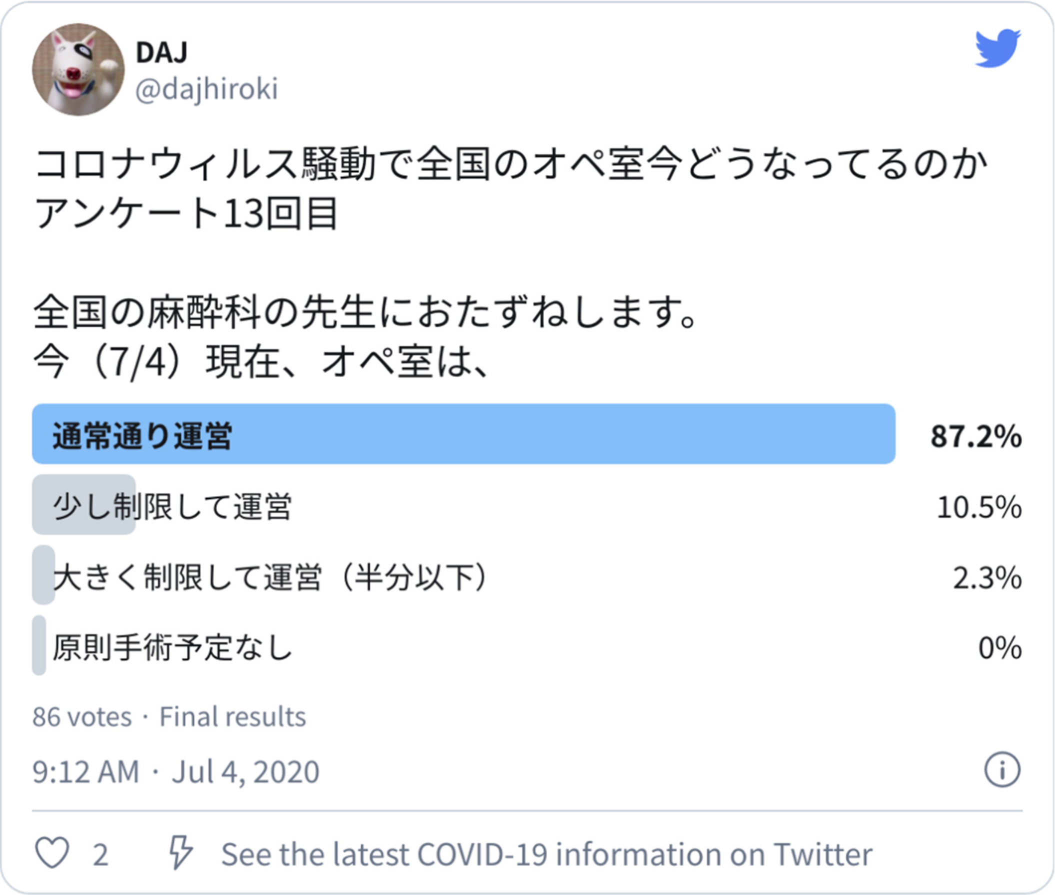

DAJ @dajhiroki

How is your operation room in coronavirus pandemic? The thirteenth trial.

I would ask anesthesiologists the question. As of 4 July (7/4), operation room performs…

Operating as usual	87.2%
Operating with partial restrictions	10.5%
Operating with extensive restrictions (less than half)	2.3%
As a general rule, no surgery	0%

https://twitter.com/dajhiroki/status/1279206368981876737

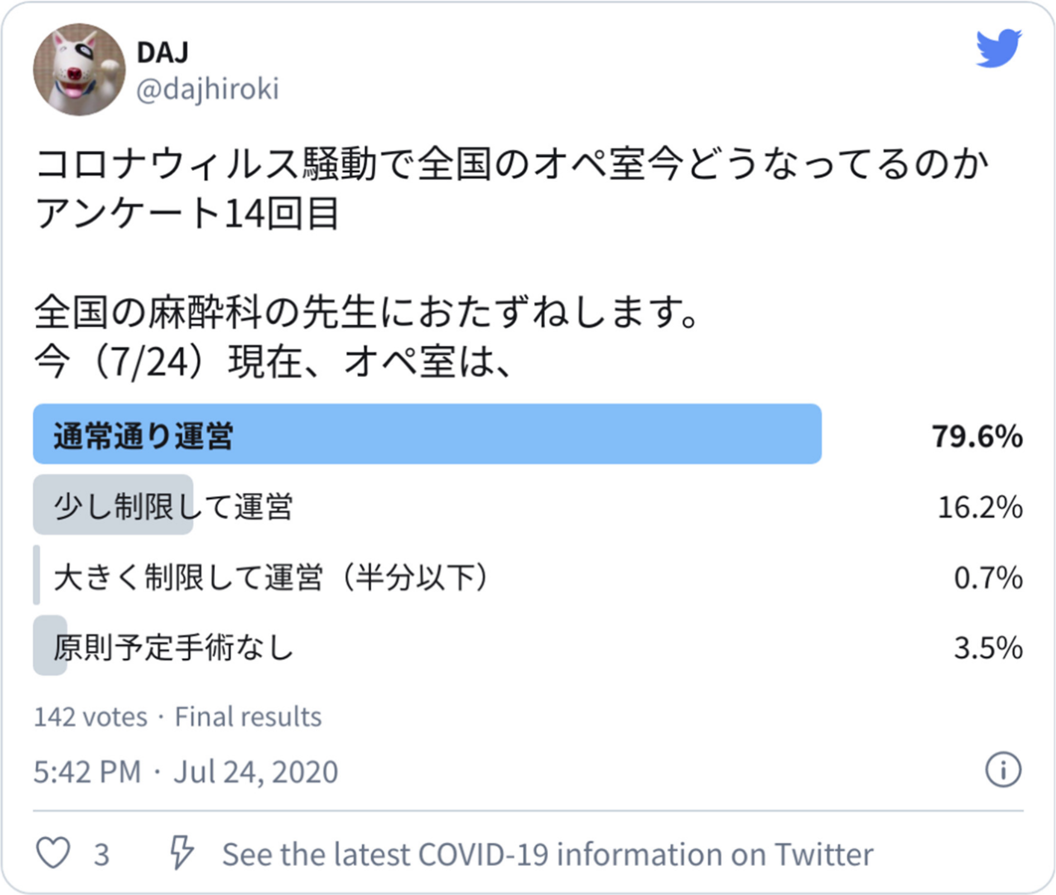
DAJ
@dajhiroki

コロナウィルス騒動で全国のオペ室今どうなってるのかアンケート14回目

全国の麻酔科の先生におたずねします。
今（7/24）現在、オペ室は、

**通常通り運営**	**79.6%**
少し制限して運営	16.2%
大きく制限して運営（半分以下）	0.7%
原則予定手術なし	3.5%

142 votes · Final results

5:42 PM · Jul 24, 2020

3 See the latest COVID-19 information on Twitter

DAJ @dajhiroki

How is your operation room in coronavirus pandemic? The fourteenth trial.

I would ask anesthesiologists the question. As of 24 July (7/24), operation room performs…

Operating as usual	76.9%
Operating with partial restrictions	16.2%
Operating with extensive restrictions (less than half)	0.7%
As a general rule, no surgery	3.3%

https://twitter.com/dajhiroki/status/1286582435774267392

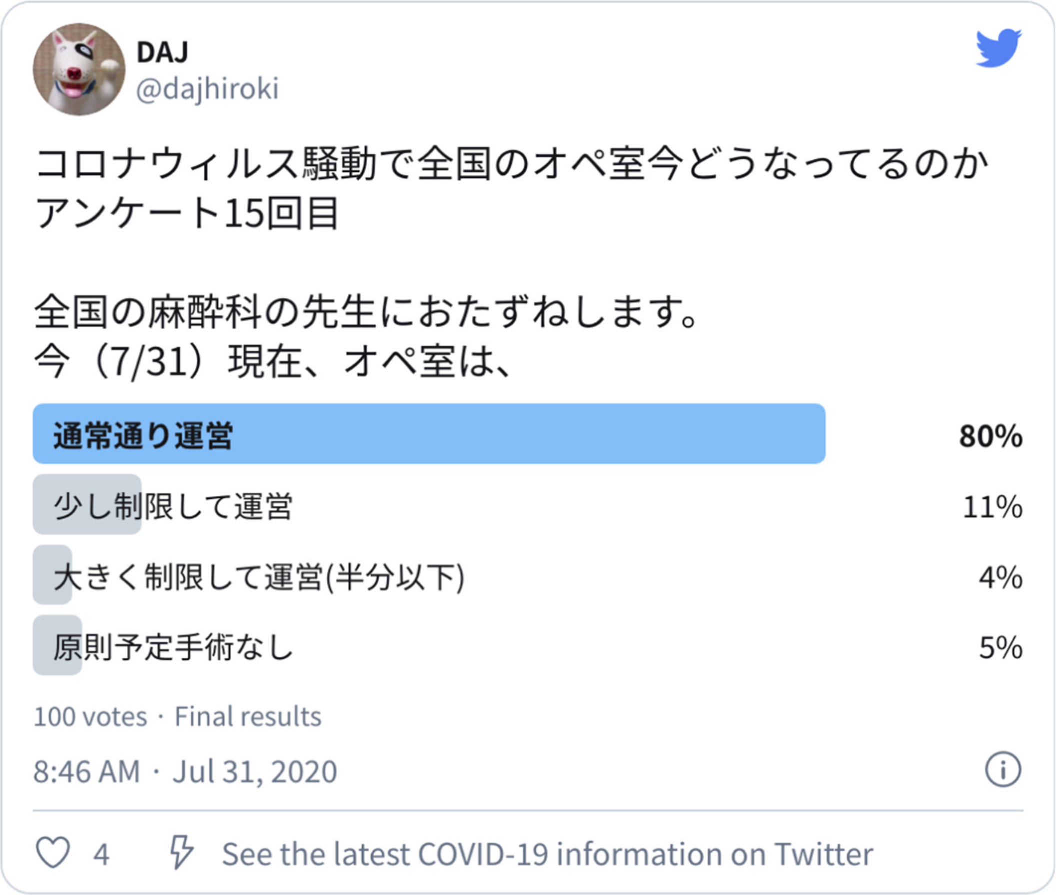
DAJ
@dajhiroki

コロナウィルス騒動で全国のオペ室今どうなってるのか
アンケート15回目

全国の麻酔科の先生におたずねします。
今（7/31）現在、オペ室は、

通常通り運営	80%
少し制限して運営	11%
大きく制限して運営(半分以下)	4%
原則予定手術なし	5%

100 votes · Final results
8:46 AM · Jul 31, 2020

4 See the latest COVID-19 information on Twitter

DAJ @dajhiroki

How is your operation room in coronavirus pandemic? The fifteenth trial.

I would ask anesthesiologists the question. As of 31 July (7/31), operation room performs…

Operating as usual	80%
Operating with partial restrictions	11%
Operating with extensive restrictions (less than half)	4%
As a general rule, no surgery	3%

https://twitter.com/dajhiroki/status/1288984358649393152

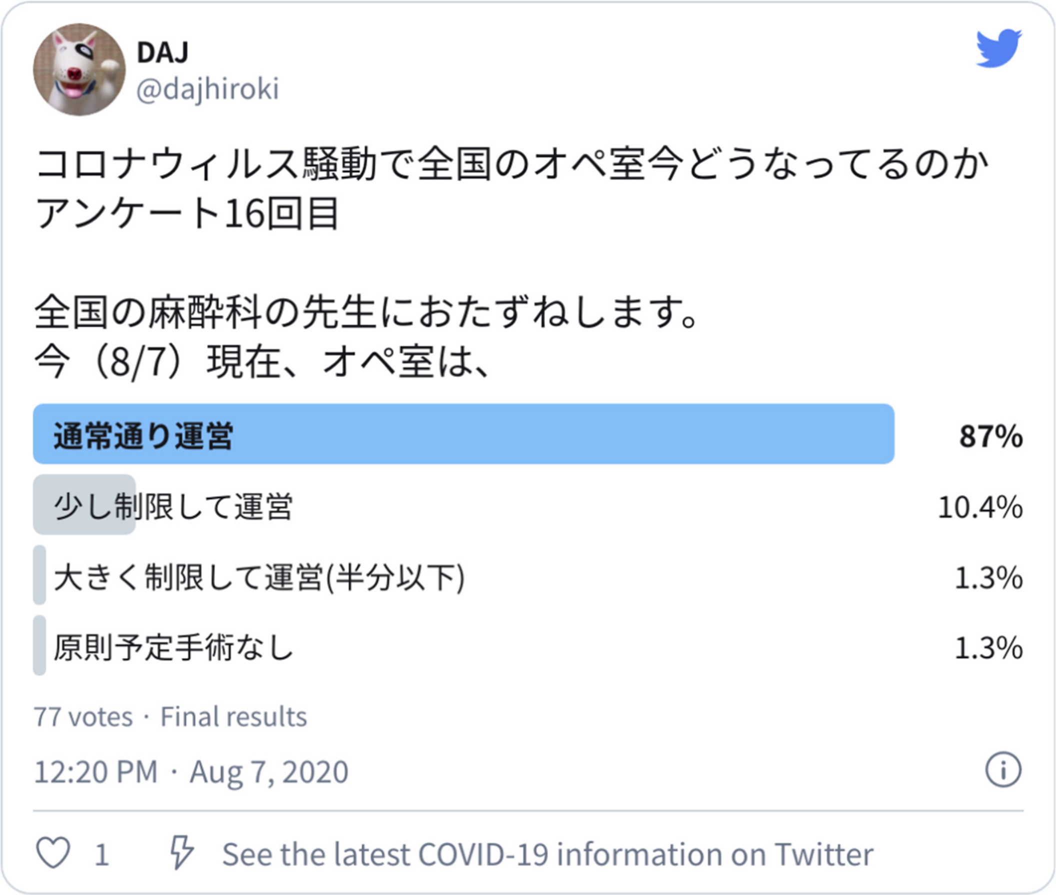

DAJ @dajhiroki

How is your operation room in coronavirus pandemic? The sixteenth trial.

I would ask anesthesiologists the question. As of 7 August (8/7), operation room performs…

Operating as usual	87%
Operating with partial restrictions	10.4%
Operating with extensive restrictions (less than half)	1.3%
As a general rule, no surgery	1.3%

https://twitter.com/dajhiroki/status/1291574839338135553

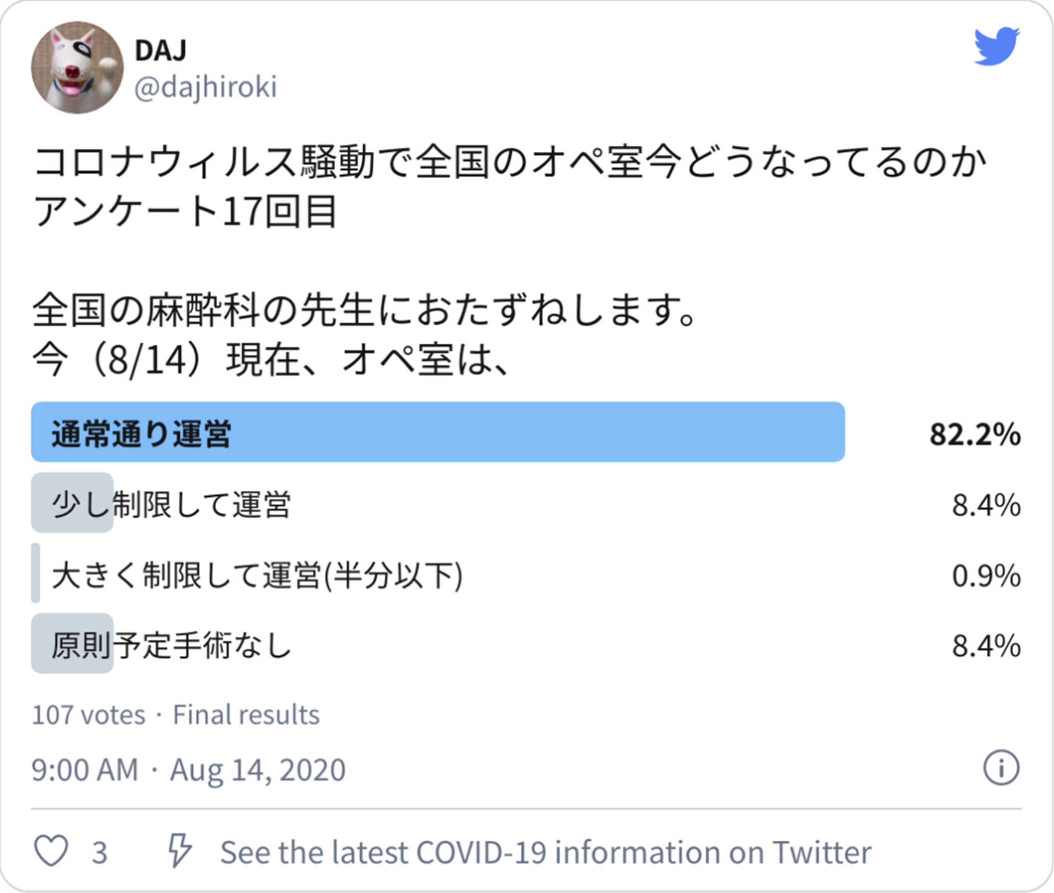

DAJ
@dajhiroki

コロナウィルス騒動で全国のオペ室今どうなってるのかアンケート17回目

全国の麻酔科の先生におたずねします。
今（8/14）現在、オペ室は、

通常通り運営	82.2%
少し制限して運営	8.4%
大きく制限して運営(半分以下)	0.9%
原則予定手術なし	8.4%

107 votes · Final results

9:00 AM · Aug 14, 2020

3 See the latest COVID-19 information on Twitter

DAJ @dajhiroki

How is your operation room in coronavirus pandemic? The seventeenth trial.

I would ask anesthesiologists the question. As of 14 August (8/14), operation room performs…

Operating as usual	82.2%
Operating with partial restrictions	8.4%
Operating with extensive restrictions (less than half)	0.9%
As a general rule, no surgery	8.4%

https://twitter.com/dajhiroki/status/1294061341154500609
